# Eczema Herpeticum and Clinical Criteria for Investigating Smallpox

**DOI:** 10.3201/eid1507.090093

**Published:** 2009-07

**Authors:** David A. Boyd, Leonard C. Sperling, Scott A. Norton

**Affiliations:** Naval Hospital Jacksonville, Jacksonville, Florida, USA (D.A. Boyd); Uniformed Services University, Bethesda, Maryland, USA (L.C. Sperling, S.A. Norton)

**Keywords:** Eczema herpeticum, smallpox, tzanck preparation, viruses, dispatch

## Abstract

Eczema herpeticum can clinically resemble smallpox. On the basis of the algorithm for rapid evaluation of patients with an acute generalized vesiculopustular rash illness, our patient met criteria for high risk for smallpox. The Tzanck preparation was critical for rapid diagnosis of herpetic infection and exclusion of smallpox.

After the 2001 anthrax bioterrorism incidents, public health officials became concerned about bioterrorist threats of smallpox. The Centers for Disease Control and Prevention (CDC), along with interested partners, developed a clinical algorithm for rapid evaluation of patients with acute generalized vesiculopustular rash illness (AGVPRI) (*1*). In a surveillance system designed to detect an index case of smallpox, high specificity is critical to minimize false-positive reports of a disease that no longer exists in nature (*2*).

CDC’s algorithm emphasizes 3 major clinical features of smallpox: febrile prodrome, typical appearance of characteristic lesions, and uniform lesion morphology (Table). The algorithm stratifies AGVPRI cases into high, moderate, and low likelihood of smallpox (*3*). Passive and active surveillance has stratified no case to high risk (*4*). We describe a patient whose illness fulfilled CDC’s high-risk criteria for smallpox, although he actually had eczema herpeticum.

**Table:** Major clinical criteria for smallpox*

Febrile prodrome	Occurring 1–4 days before rash onset; fever >101ºF; and >1 of the following: prostration, headache, backache, chills, vomiting, or severe abdominal pain.
Classic smallpox lesions	Deep-seated, firm/hard, round, well-circumscribed vesicles or pustules; as they evolve, lesions may become umbilicated or confluent.
Lesions in same stage of development	On any single part of the body (e.g., face or arm); all lesions are in the same stage of development (i.e., all are vesicles or pustules).

*Source (*3*).

## The Case

**Figure 1:**
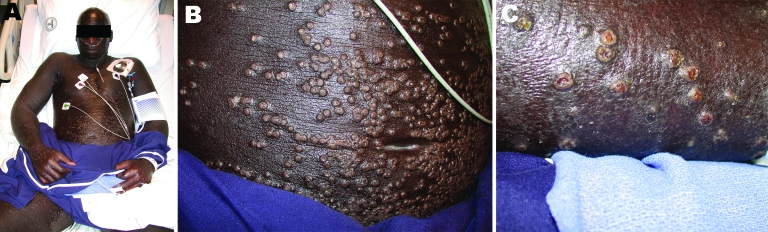
Clinical photographs of the patient. A) Patient with generalized pustules, which were deep seated, monomorphic, dome shaped, and firm and were distributed densely on forearms and abdomen. B) Umbilicated papulopustules. C) Umbilicated papulopustules in the same stage of evolution; no herpetiform clusters or red areolae are seen around the lesions.

A 45-year-old man with a lifelong history of atopic dermatitis had a year-long unremitting exacerbation for which he had started systemic therapy. After treatment with cyclosporine for several weeks, laboratory abnormalities and nonspecific neurologic signs prompted a switch to methotrexate. Within 4 weeks, he was hospitalized (in an overseas US military hospital) for generalized umbilicated papulopustules accompanied by profound hypothermia, hypotension, and mental status changes. He had large pustules on his trunk, inner thighs, and upper extremities (Figure 1, panel A). Cerebrospinal fluid, obtained because of his obtunded mental status, was unremarkable. The working diagnosis was Sezary syndrome with erythroderma. He was transferred to our intensive care unit with widespread umbilicated pustules and normal mental status. The pustules were deep seated, monomorphic, dome shaped, and firm and were distributed densely on the patient’s forearms and abdomen (Figure 1, panels B and C). He showed no enanthem or lesions with an erythematous base. Lesions were abundant on his dorsal hands, but were not palmar. His vital signs were significant only for 100.4ºF temperature. He reportedly had received vaccinia.

At our hospital, his oral temperature fluctuated dramatically, from 89.3ºF to 101.3ºF, with rectal confirmation <95ºF (<35ºC), indicating hypothermia (*5*). He remained normotensive, but his mental status fluctuated.

**Figure 2:**
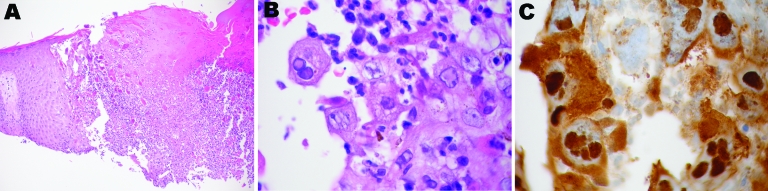
Photomicrographs of the patient’s eczema herpeticum. A) Epithelial necrosis with cellular ballooning and multinucleated giant cells. B) Ballooning degeneration of keratinocytes. C) Positive immunohistochemical stain for herpes simplex virus.

We believed this smallpox-like eruption most likely resulted from a herpesvirus. We performed a Tzanck preparation, which showed multinucleated giant keratinocytes with nuclear molding and margination (Appendix Figure). A direct fluorescent antibody (DFA) test was positive for varicella zoster virus (VZV). A biopsy specimen showed epithelial necrosis with cellular ballooning and multinucleated giant cells, plus intranuclear inclusion bodies (Figure 2, panels A and B). Subsequently, special immunohistochemical stains were positive for herpes simplex virus (HSV) (Figure 2, panel C), and a viral culture grew HSV type 2. His illness was diagnosed with disseminated HSV concurrent with underlying atopic dermatitis (i.e., eczema herpeticum).

Within minutes of the Tzanck smear evaluation, our patient was given intravenous acyclovir. When cutaneous improvement was evident, he was switched to oral valacyclovir. Within days, his skin lesions largely resolved without conspicuous crusting or scarring, but he remained intermittently hypothermic for several weeks.

## Conclusions

This patient was markedly ill on admission and had a distinctive varioliform eruption with lesions in a uniform stage of evolution. Consequently, smallpox was included in the differential diagnosis. Tzanck preparation promptly confirmed herpetic etiology, but we nevertheless used CDC’s algorithm for evaluating AGVPRI, and our patient’s illness stratified to high risk.

CDC has 3 major diagnostic criteria to designate a case as high risk for smallpox (Table) (*6*). The first is febrile prodrome, which typically lasts 1–4 days before cutaneous lesions appear and must include >1 of the following: prostration, headache, backache, chills, vomiting, or severe abdominal pain. Body temperature must reach >101ºF. Although our patient’s illness eventually met the fever criterion, his 101ºF temperature occurred only after he began antiviral treatment. He was more often markedly hypothermic during his hospitalization.

Prolonged hypothermia is associated with severe illness (*7*) and is equivalent to fever in determining critical illness (*8*), which we believe satisfies CDC’s first major criterion. The second criterion requires classic cutaneous lesions that are deep seated, firm, round, well-circumscribed vesicles or pustules that may become umbilicated or confluent. The third criterion requires the same stage for most cutaneous lesions on an affected area. Our patient’s illness met all 3 criteria; however, laboratory tests confirmed herpesvirus infection.

Smallpox was declared eradicated by the World Health Organization in 1977; nevertheless, some health organizations consider this illness a bioterrorism threat. Clinical smallpox typically starts with a prodrome of high fever, headache, myalgia, backache, nausea, vomiting, and diarrhea. An oropharyngeal enanthem is followed by cutaneous eruption of erythematous macules that quickly become papules. The papules evolve over days into vesicles and then pustules, often developing central umbilication. Classic smallpox lesions occur in the same stage of evolution on a body segment, which differentiates it from varicella. Smallpox lesions also tend to start peripherally. Smallpox pustules have been called “pearls of pus” to help distinguish them from the more delicate “dewdrops on rose petals,” which describes typical varicella. Histopathologically, cutaneous smallpox lesions may resemble herpetic lesions except that smallpox has intracytoplasmic inclusions (Guarnieri bodies) instead of intranuclear inclusions (Lipschutz bodies) of herpetic lesions. Also, multinucleated giant keratinocytes are uncharacteristic of smallpox (*9*).

Eczema herpeticum, described by Kaposi in 1887, is most common in patients with atopic dermatitis but can occur in other conditions that disrupt epidermal integrity. In eczema herpeticum, lesions are typically monomorphic vesicles that evolve into pustules (*10*). Fever, malaise, lymphadenopathy, and tender skin may accompany cutaneous eruption (*11*). The histopathologic features noted in our biopsy are classic for herpetic skin lesions.

Fever is a well-recognized sign of infection; however, hypothermia can also signal serious disease, including bacterial sepsis or viral encephalitis (*12*), and may be more dire than fever in severely ill hospitalized patients (*13*). We propose that our patient’s hypothermic temperature dysregulation is equivalent to fever, thus serving as a major diagnostic criterion.

This case shows the importance of Tzanck smears to rule out smallpox. When a patient with AGVPRI is evaluated for possible smallpox, rapid laboratory tests are necessary. Viral culture does not yield results quickly enough to avert infection control measures expected with a smallpox case. Indeed, CDC reports 7 incidents when patients with AGVPRI prompted emergency department diversions or hospital closures (*1*). Also, rapid confirmation of nonvariola etiology can help avert public panic, a potential problem in a suspected smallpox outbreak and a probable intended consequence of a terrorist attack.

The Tzanck smear must be performed by someone experienced in using the technique and interpreted by someone who can confidently and correctly distinguish herpesvivus nuclear inclusions from poxvirus cytoplasmic inclusions. DFA for HSV and VZV is relatively rapid, but in our case, the DFA result was positive for VZV, although viral culture and immunohistochemical staining later showed that the patient’s infection was due to HSV-2. Had we been unable to confirm a nonvariola etiology, we would have proceeded to poxvirus testing. With no commercially available tests for smallpox, the algorithm advises close coordination among local, state, and federal public health authorities. Some state and federal reference laboratories can provide confirmatory tests, including PCR, for orthopoxviruses such as smallpox and monkeypox. Although not performed in this case, we recommend such testing if a simultaneous infection with an orthopoxvirus cannot be ruled out.

## Supplementary Material

Appendix Figure Photomicrograph of patient's multinculeated giant keratinocytes.

## References

[1] Seward JF, Galil K, Damon I, Norton SA, Rotz L, Schmid S, Development and experience with an algorithm to evaluate suspected smallpox cases in the United States. Clin Infect Dis. 2004;39:1477–83. 10.1086/425500 15546084

[2] Centers for Disease Control and Prevention. Smallpox case definitions [cited 2008 Sept 24]. Available from http://www.bt.cdc.gov/agent/smallpox/diagnosis/casedefinition.asp

[3] Centers for Disease Control and Prevention. Evaluating patients for smallpox [cited 2007 Dec 4]. Available from http://www.bt.cdc.gov/agent/smallpox/diagnosis/pdf/spox-poster-full.pdf

[4] Hutchins SS, Sulemana I, Heilpern KL, Schaffner W, Wax G, Lerner EB, Performance of an algorithm for assessing smallpox risk among patients with rashes that may be confused with smallpox. Clin Infect Dis. 2008;46:S195–203. 10.1086/524383 18284359

[5] Danzl DF, Pozos RS. Accidental hypothermia. N Engl J Med. 1994;331:1756–60. 10.1056/NEJM199412293312607 7984198

[6] Centers for Disease Control and Prevention. Generalized vesicular or pustular rash illness protocol [cited 2007 Dec 4]. Available from http://www.bt.cdc.gov/agent/smallpox/response-plan/files/annex-4-rash-color.pdf

[7] Peres Bota D, Lopes Ferreira F, Melot C, Vincent JL. Body temperature alterations in the critically ill. Intensive Care Med. 2004;30:811–6. 10.1007/s00134-004-2166-z 15127194

[8] Levy MM, Fink MP, Marshall JC, Abraham E, Angus D, Cook D, 2001 SCCM ESICM ACCP ATS SIS International Sepsis Definitions Conference. Crit Care Med. 2003;31:1250–6. 10.1097/01.CCM.0000050454.01978.3B 12682500

[9] Slifka MK, Hanifin JM. Smallpox: the basics. Dermatol Clin. 2004;22:263–74. 10.1016/j.det.2004.03.002 15207308

[10] Wollenberg A, Zoch C, Wetzel S, Plewig G, Przybilla B. Predisposing factors and clinical features of eczema herpeticum: a retrospective analysis of 100 cases. J Am Acad Dermatol. 2003;49:198–205. 10.1067/S0190-9622(03)00896-X 12894065

[11] Stalkup JR, Yeung-Yue K, Brentjens M, Tyring SK. Human herpesviruses. In: Bolognia JL, Jorizzo JL, Rapini RP, editors. Dermatology, vol 1. Philadelphia: Mosby; 2003. p. 1235–52.

[12] Vesely DL, Mastrandrea P, Samson C, Argyelan G, Charvit S. Post-herpes encephalitic anterior pituitary insufficiency with hypothermia and hypotension. Am J Med Sci. 2000;320:273–7. 10.1097/00000441-200010000-00008 11061353

[13] Balk RA. Severe sepsis and septic shock. Definitions, epidemiology, and clinical manifestations. Crit Care Clin. 2000;16:179–92. 10.1016/S0749-0704(05)70106-8 10768078

