# Supplementary figures and images for: Eczema Herpeticum and Clinical Criteria for Investigating Smallpox
Source: Emerg Infect Dis. 2009 Jul;15(7):1102–4. doi: 10.3201/eid1507.090093 (PMC2744234; doi:10.3201/eid1507.090093)

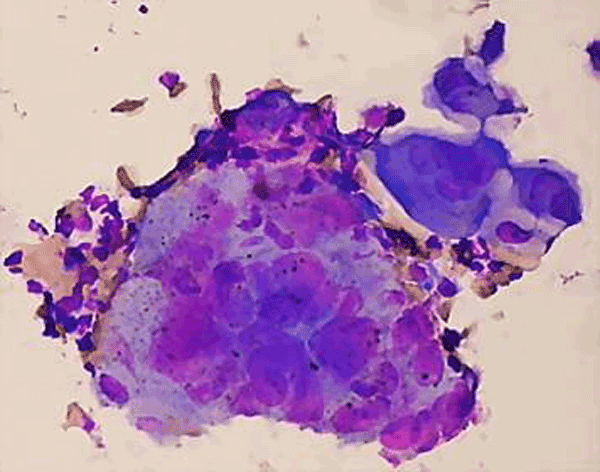

Supplement: Appendix Figure — Photomicrograph of patient's multinculeated giant keratinocytes. [file 09-0093_appF-s1.gif]
